# Do Health Literate Older People Have Fewer Depressive Symptoms? Evidence from the Urban Areas of Western China

**DOI:** 10.3390/healthcare12232373

**Published:** 2024-11-26

**Authors:** Chuchen Huang, Weixiu Cui, Ye Yang, Xinlin Huang, Chengbo Li, Ziyue Lin

**Affiliations:** School of Journalism and Communication, Chongqing University, Chongqing 400044, China

**Keywords:** health literacy, depressive symptoms, older Chinese adults

## Abstract

Background: Previous research has indicated that health literacy has a good influence on the mental health among older adults. The current study aims to determine whether health literacy is linked to depressive symptoms in older adults in west China, and tries to detect the mechanisms underlying the linkage between health literacy and depressive symptoms in the Chinese context. Methods: Study data were obtained from a cross-sectional ageing health literacy survey conducted in 2017 in Western China, and 812 urban individuals aged 60 and older were selected. Results: Health literacy was associated with fewer symptoms of depression; additionally, older adults who reported harmonious family and filial piety, those with greater self-rated health, and those with a higher level of life satisfaction tended to have fewer depressive symptoms, whereas older individuals who reported a poor financial status and those with functional impairment had more depressive symptoms. Conclusions: The findings indicate the protective role of health literacy against depressive symptoms among urban older adults. In addition, the findings also indicate the potential impact of financial strain and functional impairment on the development of depressive symptoms and show that other factors including a harmonious family, filial piety, self-rated health and life satisfaction are generally protective against depressive symptoms. Specific evidence is presented for the national action plans and public health strategies needed to reinforce and promote health literacy in the Chinese context. The current results also suggest that health education and promotion programs designed to strengthen financial assistance, functional improvement, family relationships, life satisfaction and self-rated health should gain a growing importance on the health agenda. Future longitudinal studies, mediation or moderator models, and structural equation modeling would be needed to identify a causal relationship, the moderating or mediating effect, and the possible pathways underlying the linkage between health literacy and depressive symptoms, respectively.

## 1. Introduction

Health literacy is defined as the ability to obtain, understand and use health-related information and services to make well-informed decisions, and to know how to evaluate them [1]. However, a lack of health literacy is particularly prevalent among older Chinese adults. In China, a vast majority of older adults have an inadequate level of health literacy; of those aged 65~69, approximately 91.51% of older adults and 91.45% of older people in urban environments were considered to be health illiterate in 2020 [2] and in 2019 [3], respectively. In addition, a meta-analysis study also showed that 87.72% of older adults were health illiterate in China in 2021 [4]. In order to bring older adults’ health literacy up to an adequate level, the Chinese government has implemented a national health literacy reinforcement program in the Health China Action Plans (2019–2030) [5].

Depression is defined as a prevalent and burdensome psychiatric disorder [6,7,8], and is an important cause of disease burden in the world [9,10]. With an aging population, depression is an insidious health problem faced by older adults [8]. A relatively large proportion of old people are suffering from depressive symptoms worldwide. One meta-analysis study indicated that the prevalence of depression is approximately 28.4% among older adults aged 60 and above at the global level [11]. In a longitudinal study, 23.02% of older people aged 65 and above were depressed in 2018 in China [12]. There is evidence that depressive symptoms usually have a serious impact on older adults [8,13,14,15]. For instance, it can break social relations [8], exert a huge burden on caregivers [16], reduce patients’ quality of life [8] and present challenges to the healthcare system [17]. Moreover, depressive symptoms are not often well recognized, highlighted and treated, because they co-occur with other health problems and resemble the aging process in older adults [13]. 

Given the lower prevalence of health literacy older people face, it is important to explore how health literacy influences depressive symptoms among older adults in China. Despite the lower prevalence of health literacy in older populations, only a few studies have evaluated the influence of health literacy on older adults’ mental health. Previous studies have shown that the reinforcement of health literacy must be considered an important priority to prevent depressive symptoms [18,19,20,21,22,23,24]. Health literacy has been associated with a reduction in depressive symptoms among adults in Korea [20], Japan [25] and Vietnam [21], and is particularly linked to a reduction in depressive symptoms among older Vietnamese adults [22]. Specifically, being health illiterate is related to an increase in depressive symptoms. As a risk factor, a lower level of health literacy is independently linked to depression, above and beyond the socioeconomic status factors observed in smokers with a low socioeconomic status [26]. Patients with lower health literacy could contribute to a greater burden of depressive symptoms in Europe [19], Sweden [23], Chengdu, China [27] and the U.S. [28]. A higher level of health literacy is particularly linked to a reduction in depressive symptoms in differentiated thyroid carcinoma patients in west China [18].

However, few studies have thoroughly assessed a model of the various ways that social determinants and health status factors might operate in terms of influencing the linkage between health literacy and depressive symptoms. In previous studies, efforts have been directed toward understanding the role that social determinants and health status play in personal depressive symptoms. Social determinants and health status include four main factors: demographic and socio-economic characteristics, family relationships and health status. Age positively influences depressive symptoms among older individuals, and depressive symptoms are particularly common in older adults across the world [29]. The positive relationship between age and depression has been found among older adults in China [17,30] and Turkey [7]. In comparison to their counterparts, older females appear to be more depressed; such a correlation could be applicable to both China [31] and the U.S. [32]. Past studies have discovered that a higher level of depression is prevalent among older individuals with a lower socioeconomic status [17]. Educational attainment is correlated with depression. However, the direction of the relationship remains controversial [33]. Some studies have shown that higher education levels are associated with a reduction in depressive symptoms among older adults in China [30] and other countries including Ghana, India, Mexico, South Africa and Russia [15,34]. Some research has shown that higher education levels have a significant positive association with depression in adults [21,33]. Financial strain is an important determinant of geriatric depression; specifically, older people who have a lower income show a higher prevalence of depression in Turkey [7] and India [35]. Occupation is shown to be linked to depression, as older individuals with past informal occupations are more likely to have a higher prevalence of depression [15]. The risk of depression is significantly higher in the blue-collar and sales and service worker groups than the white-collar group [36]. Professionals have a negatively significant association with depression, whereas business owners have a positively significant association with depression [37]. A good family relationship is important for depression prevention among older adults [30,38]. Harmoniousness could benefit older people by alleviating late-life depression in the family. Filial piety is a unique and essential factor that contributes to a reduction in depressive symptoms in older people in China [39].

Poor self-rated health and physical limitations are essential factors that contribute to the odds of depression in older individuals [40] and are particularly negatively associated with depression among older Chinese adults [31] and Korean adults [20]. The literature has identified that suffering from chronic diseases could result in depression among older adults [13,14]. Some systematic reviews have proposed that functional impairments are important predictors that could reinforce depression in older adults [14,40]. In China [41], Korea [42], Brazil [43] and Turkey [8], life satisfaction has been shown to negatively impact depressive symptoms.

Drawing from the relevant work above, we can find that many studies have explored the social determinants and health status factors associated with depressive symptoms. However, as these social determinants and health status factors are inter-correlated, it is necessary to control possible covariates to give a better evaluation of the direct effect of health literacy on depressive symptoms. At the same time, we can identify two articles involving the linkage between health literacy and depression [18,27] in China. However, the two studies were limited to specific older patients with specific chronic diseases, and they relied on samples from specific regions in Chengdu. Therefore, previous work focused on detecting the linkage between health literacy and depressive symptoms remains scarce in the Chinese context. Therefore, based on the overall older population and representative data, and by controlling possible covariates, our goal is to focus on detecting the linkage between health literacy and depressive symptoms among urban older adults in Western China. The rest of the paper includes five sections: research methods, results, discussion, implications and limitations, and conclusions.

## 2. Research Method

### 2.1. Data

Study data were collected from a cross-sectional ageing health literacy survey that was conducted from July to September in 2017 in Western China, and 812 urban individuals aged 60 and older were selected [44,45,46]. The survey covered a representative sample of regions such as Yinchuan in Ningxia, Wenshan in Yunnan, and Yongchuan in Chongqing. 

**Figure 1:**
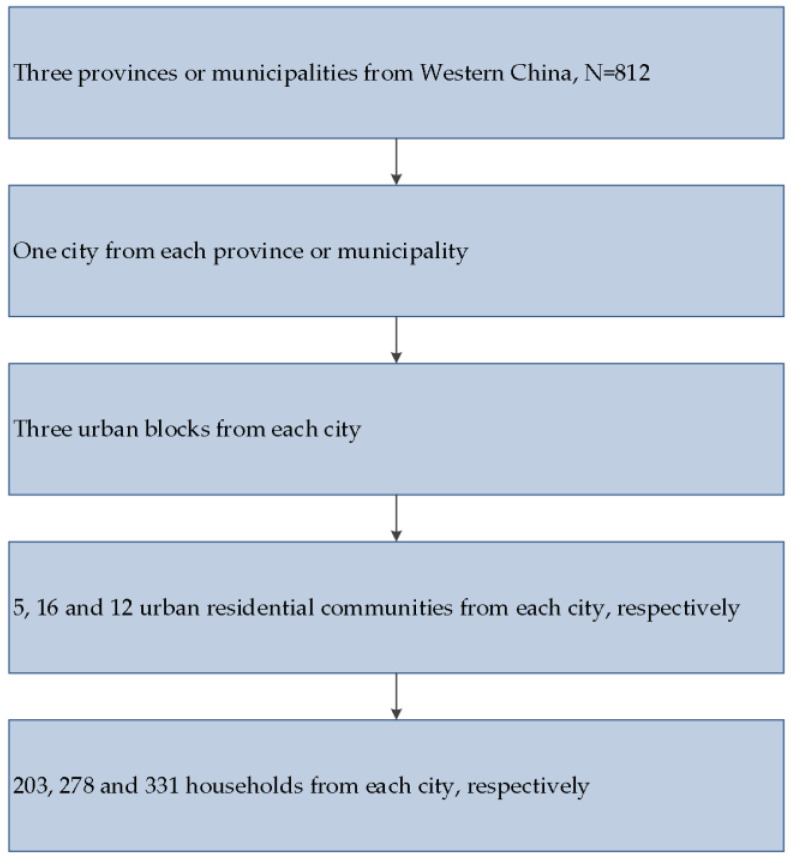
The flowchart.

We used cluster sampling in the present survey; as the following flowchart (Figure 1) shows, the procedures included five stages. In the first stage, to ensure all geographical regions (southwest and northwest) were appropriately represented, three administrative divisions (Ningxia, Yunnan and Chongqing) were selected from all provinces or municipalities in China’s western region, and we selected Ningxia municipality in China’s northwestern region, and Yunnan province and Chongqing municipality in China’s southwestern region. In the second stage, we selected Yinchuan city in Ningxia, Wenshan city in Yunnan, and Yongchuan district in Chongqing. In the third stage, we selected three urban blocks from three cities, namely Yinchuan, Wenshan, and Yongchuan. In the fourth stage, we selected a total of 5, 16 and 12 urban residential communities from the three blocks in Yinchuan, Wenshan and Yongchuan, respectively. In the fifth stage, households were selected from the selected urban residential communities, resulting in data for 812 urban households, including 203, 278 and 331 households in Ningxia, Yunnan and Chongqing, respectively. If one household contained multiple members aged 60 or above, one member would be chosen using the Kish table.

In addition, we used a health literacy questionnaire and face-to-face personal interviews to implement the survey. The data collection was conducted in a responsible manner with the written consent of the interviewees.

### 2.2. Variables

In our study, the dependent variable was depressive symptoms. The independent variable was health literacy. The controlled attributes included demographic variables (gender and age), socio-economic information (educational level, occupation, financial strain), family relationships (family harmony and filial piety), and health status (self-rated health, suffering from chronic diseases, functional impairment and life satisfaction).

### 2.3. Measurement Instruments

#### 2.3.1. Depressive Symptoms

Depressive symptoms were assessed using an abbreviated 15-item Geriatric Depression Scale (GDS-15) [47], which ensured good reliability and validity for identifying mental health among older adults [45]. The participants were asked whether they had experienced certain feelings or behaviors during the one-week period preceding the interview [48]. Among the 15 items, 10 items assessed negative affect and behavior (dropping many activities and hobbies; feeling that life is empty; often getting bored; afraid that something bad is going to happen; feeling helpless; having nothing to do; feeling increasingly forgetful; feeling not helpful; feeling hopeless; thinking that most people are better off), and five items assessed positive affect (basic satisfaction with life; in good mood most of time; feeling happy; thinking it is wonderful to be alive now; feeling full of energy). For each item, 0 represented no, while 1 represented yes; the scores for depressive symptoms ranged from 0 to 15. In our sample, Cronbach’s alpha for the 15 items was 0.802.

#### 2.3.2. Health Literacy

In our study, health literacy was a composite term, definition and conceptual framework. Based on this evidence, the China Health Educational Center developed the Chinese version of the questionnaire to assess health literacy. This questionnaire is a standard, multidimensional and comprehensive measurement tool, especially for older adults, and it was applicable, reliable and valid for describing the ability of people to meet complex demands related to health in the past literature about China [45,49,50,51,52,53]. Moreover, the Chinese version of the questionnaire included 80 items associated with health literacy: 38 for the health knowledge domain, 22 for the health behavior domain and 20 for the health skills domain. A detailed statement of the 80 items for health literacy was further provided by Nie et al. [53] and Li. [52]. The internal consistency reliability for health literacy was high in the current sample (Cronbach’s alpha = 0.937). 

The format of all the test items was changed to four types of questions, which consisted of true or false questions, single-select multiple-choice questions (each correct answer scored one point and each incorrect answer scored zero points), multi-select multiple choice questions (each correct answer scored two points and each incorrect answer scored zero) and situational interview questions. Specifically, with common and relevant medicine and health information, instructions and knowledge in everyday life, certain reading comprehension statements were well designed for the situation questions, and the format here was changed from single-answer questions to multiple-answer questions, and the scoring criteria here was exactly same as the single or multiple-answer questions described above. In each item, the answers to the question were well divided into certain categories, and an answer category of “I don’t know” was added; this was given a score of zero. The overall scores were computed by adding the scores for the 80 items with equal weighting [53,54], and the scores for health literacy ranged from 1 to 94 in the present research. 

#### 2.3.3. Other Variables

Demographic variables included gender and age. For gender, 0 represented males while 1 represented females. For age, those who were between 60 and 64 years old were coded as 1, those between 65 and 69 years old were coded as 2, those between 70 and 74 years old were coded as 3, those between 75 and 79 years old were coded as 4, and those aged 80 and above were coded as 5. 

Socio-economic status included educational attainment, occupation, and financial strain. Educational attainment was a 5-response categorial variable (1 = illiterate, 2 = primary school, 3 = junior high school, 4 = polytechnic school or senior high school, 5 = college and above). Occupation was classified into six main categories; specifically, “ordinary staff” was coded as 1, “professional” was coded as 2, “manager” was coded as 3, “service industry employee” was coded as 4, “production staff” was coded as 5, and “other” was coded as 6. For financial strain, it was measured by a single question: “How do you report your financial strain condition now?”. Respondents were asked to rate this item on a five-point Likert scale (1 = more than sufficient, 2 = good sufficient, 3 = approximately sufficient, 4 = somewhat difficult, 5 = very difficult). Due to the severe skewness of the distribution of percent in our current research, we collapsed this variable into three categories: sufficient (more than sufficient and good sufficient), approximately and difficult (somewhat difficult and difficult).

Family relationships comprised family harmony and filial piety. For family harmony, respondents were asked whether their family was harmonious; this study used a dummy variable “family harmony” (0 = disharmonious, 1 = harmonious). For filial piety, respondents were asked whether their children followed filial norms, and responses were scored on a five-point Likert scale (not at all = 1, not very much = 2, fair = 3, fairly = 4, very much = 5).

Health status comprised self-rated health, suffering from chronic diseases and life satisfaction. For self-rated health, participants were asked a single item: “How do you assess your health situation now?” Then, their responses were scored on a 5-point Likert scale (1 = very bad, 2 = bad, 3 = fair, 4 = good, 5 = very good). Due to the severe skewness of the distribution of percent in the present study, we collapsed this variable into three categories: bad (very bad and bad), fair and good (good and very good). We used a single item to measure suffering from chronic diseases, and this item included a single question: “Do you suffer from any chronic disease? (0 = no, 1 = yes)”. 

In our sample, functional impairment was measured using the activities of daily living (ADL) and the instrumental activities of daily living (IADL) scale. The ADL is a five-item scale that assesses a person’s ability to bathe, dress, go to the toilet, transfer from bed to chair, and eat. IADL is a six-item scale that assesses a person’s capacity to use a telephone, travel via car or public transportation, go shopping for food or clothes, prepare a meal, perform housework, and manage money. For the eleven items, responses to the measure were divided into 1, for having no difficulty, 2, for having a little bit of difficulty, and 3, for having difficulty in performing these tasks independently. The scores of ADL and IADL ranged from 11 to 33, and higher scores indicated poorer functional ability. The internal consistencies of the ADL and IADL measures were 0.89 and 0.88, respectively, which were high-level values.

Life satisfaction was assessed using a single item: “Overall, how do you rate your current life? (1 = very dissatisfied, 2 = fairly dissatisfied, 3 = fair, 4 = fairly satisfied and 5 = very satisfied)” [48]. Due to the severe skewness of the distribution of percent in the present study, we collapsed this variable into three categories: dissatisfaction (very dissatisfied and fairly dissatisfied), fair and satisfactory (fairly satisfied and very satisfied).

### 2.4. Analytic Strategy

First, descriptive analyses were conducted with dependent variables and independent and controlled variables. Second, we established two linear regression models to observe the influence of health literacy on depressive symptoms. The first linear regression analysis—with health literacy introduced as an independent variable—was conducted to assess the association between health literacy and depressive symptoms. The second linear regression analysis was applied to control the other factors including demographic and socio-economic information, family relationships and health status to better test the association between health literacy and depressive symptoms. IBM SPSS 25.0 software (SPSS Inc., Chicago, IL, USA) was used in the present data analysis. 

## 3. Results

### 3.1. Characteristics of the Sample 

Table 1 presents the characteristics and level of depressive symptoms according to different characteristics among the participants. The mean of depressive symptoms was 3.01, and the overall average health literacy score was 56.32. Moreover, significant differences in depressive symptoms existed in the present sample in terms of educational level, occupation, financial strain, harmonious family, self-rated health, suffering from chronic diseases and life satisfaction. We also found a significant correlation between depressive symptoms and the three continuous variables, namely health literacy, functional impairment and filial piety.

### 3.2. Linear Regression Model

For Table 2, linear regression model I—with health literacy introduced as an independent variable—explained 12.3% of the variance in depressive symptoms. Among the participants, health literacy (B = −0.057, *p* < 0.001) was significantly associated with the reduction in depressive symptoms. Specifically, participants who reported higher levels of health literacy had lower depressive symptom scores.

As presented in Table 3, after adjusting for all the controlled variables, we established a new linear regression model to identify whether health literacy was still significantly associated with depressive symptoms. First, we checked the multicollinearity among all potential variables in the regression model. All the values of tolerance (>0.1) and VIF (<5) met the multicollinearity criteria. In other words, no multicollinearity remained in the regression.

Second, we established a new linear regression model, which incorporated all the controlling factors including demographic attributes, socio-economic status, family relationships and health status. With strong predictive power (R^2^ = 0.482), the current regression model could further and better demonstrate the impact of health literacy on depressive symptoms.

Among the respondents, health literacy (B = −0.012, *p* < 0.05) was associated with fewer depressive symptoms; additionally, older adults who reported a harmonious family (B = −1.520, *p* < 0.001) and filial piety (B = −0.365, *p* < 0.001), those with greater self-rated health (B = −1.306, *p* < 0.001), and those with a higher level of life satisfaction (B = −4.259, *p* < 0.001) tended to have fewer depressive symptoms, whereas older individuals who reported a poor financial status (B = 0.985, *p* < 0.001) and those with functional impairment (B = 0.070, *p* < 0.05) had more depressive symptoms (Table 3). Specifically, respondents who obtained higher levels of health literacy had lower depressive symptom scores. Respondents whose self-rated health was “fair” and “good” and whose life satisfaction was “fair” and “satisfactory”, and those with a harmonious family and greater filial piety, had lower depressive symptom scores. Respondents who reported a financial strain status of “difficult” and those with functional impairment had higher depressive symptom scores.

## 4. Discussion

The findings of the present study indicated the direct linkage between health literacy and depressive symptoms, highlighting the effect of health literacy on reducing depressive symptoms among older adults living in urban environments in Western China; similar results were found in prior research [20]. The reason for this inhibitive influence was that health literacy concerns individuals’ capacities to meet the complex demands of health in modern society [55], that it was an important determinant of health [56], and that it might benefit individuals in achieving good health outcomes [57].

As a contributing factor, health literacy could act as knowledge, a belief, a tool, a skill and asset that encourage people to access to mental health education, counseling and training, and utilize existing help, programs and support groups to promote, protect and restore mental health [20,27,58]. Specifically, individuals with a higher level of health literacy would have obvious strengths, including higher health awareness [59], multiple sources of health information and sufficient health information [60,61,62] and greater intentions to share health-related information [60], which could be beneficial to help-seeking intentions [59] and improve mental health literacy [63]; this in turn could play a protective role in the recognition, management, or prevention of mental health symptoms [64].

However, a lower level of health literacy is associated with an increase in depression. Due to the shame of being health illiterate, older people might have lower intentions to share health-related information and hide their health-related problems from others, thereby resulting in isolation, less available support [27,65] and a depressed mood [26].

In addition to the linkage between health literacy and depressive symptoms, we can also confirm the influence of controlling possible covariates including financial difficulty, a harmonious family, filial piety, self-rated health, functional impairment and life satisfaction on depressive symptoms among older adults.

The current findings indicated that an increase in financial difficulty would lead to an increase in depressive symptoms. Why did financial strain exert a deleterious influence on depressive symptoms among older adults? This might be because financial difficulty usually referred to the bad conditions in which old people were born, have grown, worked, lived, and aged during their lifetime; this includes, for example, bad nutrition or malnutrition, or being denied access to better healthcare services [7]. These bad conditions could be extremely damaging to older adults’ mental health [15], and then result in a depressive state.

Harmonious family, as an inhibitive factor, played an important role in depression among older adults. Why did harmonious family affect depressive symptoms? This might be because a harmonious family is be a source of support [66]; in harmonious families, financial, instrumental, emotional and information support could be provided for older individuals, while a disharmonious family would be a source of stress and lead to a lack of support in the family; this was a risk factor related to depressive symptoms [67]. 

The present study’s findings highlighted the influence of filial piety on depressive symptoms in older adults. The reason for this influence was that filial piety has remained active in Chinese culture and the societal context; it was important to the family and older adults, and beneficial to their mental health [30]. Inversely, neglecting filial duties was regarded as inhuman and immoral; it would have serious consequences for older individuals, and result in negative effects such as depressive symptoms.

These findings also highlighted the significant association between self-rated health and depressive symptoms among older adults. The influence of self-rated health on depression was confirmed by prior studies [31,33,68]. From a psychological point of view, this influence might be explained by the psychological mechanisms underlying the self-assessment of one’s health. Individuals might perceive a threat to their self-integrity and self-worth when they report their self-rated health as “bad”, and this could increase their negative affect [69] and depressive state. Conversely, this perceived threat to health did not exist when people reported their self-rated health as “fair” and “good”, possibly contributing to the prevention of depressive symptoms.

Our findings confirmed the protective role of life satisfaction against depression in older adults. Similar findings were observed in other studies conducted in China [41], Korea [42], Brazil [43] and Turkey [8]. Why was life satisfaction negatively related to depression? A mechanism through which life satisfaction could impact depressive symptoms might be that life satisfaction could help protect against negative affect, then contribute to a reduction in depressive symptoms among older adults [8,41]. The improvement of mental well-being variables like life satisfaction was generally protective against mental illness such as depression [70,71,72].

Older individuals with functional impairment were found to be more likely to suffer from depression, which was similar to the results of previous studies [14,40]. There were two possible reasons for the positive relationship between functional impairment and depressive symptoms. First, functional impairment was often treated as evidence for the increasing chances of depression. Secondly, older adults with functional impairment take part in fairly restricted activities of daily living and instrumental activities of daily living, which might directly result in a heavy burden of care in the family, and in turn lead to psychological strain and feelings of guilt, causing feelings of depression. 

## 5. Implications and Limitations

### 5.1. Implications

The current study indicated the influence of health literacy on depressive symptoms. In light of this, national action plans and public health strategies are needed to promote health literacy, as suggested by the China government [5], to prevent and reduce depressive symptoms. Efforts and procedures must be made to deliver effective preventive interventions and improve our ability to identify and treat depressive symptoms among older adults. First, mental health professionals and services should provide regular mental health education, counseling and training, raising mental health awareness for older individuals. Secondly, health practitioners, community workers and family members should encourage older adults to actively participate in help-seeking activities for the recognition, management, or prevention of mental health symptoms. Thirdly, community workers and family members should enhance older adults’ intentions and behavior, and encourage them to share health-related information and problems.

Moreover, relying on the evidence that the six controlling variables including financial strain, family harmony, filial piety, self-rated health, functional impairment and life satisfaction were associated with depressive symptoms, it is possible that three strategies are needed for preventing depressive symptoms. First, it could be particularly useful to foster harmony and support filial piety to build good and healthy relationships in the family and community. Secondly, the issues of financial assistance and functional improvement should be considered simultaneously in the design, implementation and development of all health and social service provisions in health policies. Thirdly, the integration of health promotion programs designed to strengthen life satisfaction and self-rated health deserves attention in prevention efforts and strategies; moreover, there is evidence that life satisfaction should be treated as a key component in the reduction in depressive symptoms among older adults.

### 5.2. Limitations

This study had its limitations. First, the data used had limitations. Due to the COVID-19 epidemic, millions of people worldwide have been affected by rapid and dramatic social change in recent years. We failed to consider the effect of the COVID-19 epidemic on mental health such as depressive symptoms among older adults in the present data and research. 

Second, due to the cross-sectional design of the current research, we failed to detect the causal association between health literacy and depressive symptoms. Future longitudinal studies would be needed to identify a causal relationship between health literacy and depressive symptoms among older adults. 

Third, the current linear regression models had two limitations. On the one hand, the moderating or mediating effect on the linkage between health literacy and depressive symptoms was not considered in the present analysis. By applying a mediation or moderator model, future studies could evaluate the impact of mediator or moderator variables on the effect of health literacy and depressive symptoms. More specifically, suffering from chronic disease, as a mediating variable, could be introduced and examined in future studies. On the other hand, previous findings indicated the reciprocal relationships between life satisfaction and depression [73]. However, the present study did not examine the possible pathways between depressive symptoms and life satisfaction. In future, the possible pathways could be determined through structural equation modeling. 

## 6. Conclusions

Our current findings highlight the ability of health literacy to protect against depressive symptoms among urban older adults in Western China. In addition, the present findings also indicate the potential impact that financial strain and functional impairment have on developing depressive symptoms and show that other factors, including harmonious family, filial piety, self-rated health and life satisfaction, are generally protective against depressive symptoms. Based on these findings and their implications, specific evidence is presented for the national action plans and public health strategies needed to reinforce and promote health literacy in the Chinese context. Efforts and procedures must be made to deliver effective preventive interventions and improve our ability to identify and treat depressive symptoms among older adults. The current results also suggest that health education and promotion programs designed to strengthen financial assistance, functional improvement, family relationships, life satisfaction and self-rated health should be important on the health agenda. Future longitudinal studies, mediation or moderator models, and structural equation modeling would be needed to identify causal relationships, moderating or mediating effects, and the possible pathways underlying the linkage between health literacy and depressive symptoms, respectively.

## Figures and Tables

**Table 1:** Characteristics and level of depressive symptoms according to different characteristics among the participants (N = 812).

Categorical Variables	N (%)	Level of Depressive Symptoms		
		Mean (SD)	*F*/*t*	*p*
Gender			−0.872 ^a^	0.383
Male	378 (46.6)	2.915 (2.694)		
Female	433 (53.4)	3.097 (3.235)		
Age			1.276 ^b^	0.278
60–64	220 (27.2)	2.805 (2.921)		
65–69	187 (23.1)	2.888 (2.806)		
70–74	186 (23.0)	3.280 (3.345)		
75–79	121 (15.0)	2.868 (3.157)		
80+	95 (11.7)	3.442 (2.546)		
Education level			19.161 ^b^	<0.001
Illiterate	165 (20.3)	4.485 (3.598)		
Primary school	247 (30.5)	3.271 (3.102)		
Junior high school	235 (29.0)	2.430 (2.340)		
Polytechnic school or senior high school	116 (14.3)	2.000 (2.269)		
College and above	48 (5.9)	1.917 (2.386)		
Occupation			10.046 ^b^	<0.001
Ordinary staff	55 (6. 8)	2.018 (1.861)		
Professionals	154 (19.0)	1.799 (1.887)		
Manager	15 (1.8)	1.800 (2.111)		
Service industry employee	65 (8.0)	3.169 (3.100)		
Production staff	465 (57.3)	3.514 (3.212)		
Others	57 (7.0)	3.298 (3.375)		
Financial strain			106.397 ^b^	<0.001
Sufficient	334 (41.2)	1.952 (2.160)		
Approximately	334 (41.2)	2.868 (2.584)		
Difficult	143 (17.6)	5.825 (3.725)		
Harmonious family			8.245 ^a^	<0.001
Not harmonious	38 (4.8)	7.868 (3.807)		
Harmonious	761 (95.2)	2.714 (2.674)		
Self-rated health			74.619 ^b^	<0.001
Bad	110 (13. 6)	5.473 (3.734)		
Fair	280 (34.5)	3.507 (3.028)		
Good	421 (51.9)	2.040 (2.211)		
Suffering from chronic diseases			−5.243 ^a^	<0.001
No	130 (16.1)	1.977 (2.338)		
Yes	679 (83.9)	3.217 (3.067)		
Life satisfaction			253.388 ^b^	<0.001
Dissatisfaction	67(8.3)	8.672 (3.164)		
Fair	90 (11.1)	4.622 (2.625)		
Satisfaction	654 (80.6)	2.211 (2.210)		
**Continuous Variables**	**N**	**Mean (SD)**	**Correlation with Depression**	***p***
Health literacy (range: 1–94)	812 (100.0)	56.32 (18.44)	−0.352	<0.01
Functional impairment (range: 11–33)	812 (100.0)	11.94 (2.51)	0.172	<0.01
Filial piety (range: 1–5)	812 (100.0)	4.31 (0.81)	−0.370	<0.01
Depressive symptoms (range: 0–15)	812 (100.0)	3.01 (2.99)	-	-

Abbreviation: ^a^ represented for *t* value; ^b^ represented for *F* value.

**Table 2:** Linear regression model I regarding health literacy linked to depressive symptoms.

Predictors	B (95% CI)	β	*p* Value
Health literacy	−0.057 (−0.068, −0.047)	−0.352	0.000
Constant	6.231 (5.609, 6.852)		0.000
Adjusted R Square	0.123		
*F*	114.458		
*p*	0.000		

**Table 3:** Linear regression model II regarding health literacy linked to depressive symptoms.

Predictors	B (95% CI)	β	*p* Value	Tolerance	VIF
Health literacy	−0.012 (−0.025, −0.000)	−0.077	0.046	0.438	2.286
Gender					
Male (reference)					
Female	−0.17 (−0.483, 0.139)	−0.029	0.278	0.908	1.101
Age with 5 years increment	−0.01 (−0.137, 0.110)	−0.006	0.834	0.798	1.253
Education level					
Illiterate (reference)					
Primary school	−0.25 (−0.730, 0.221)	−0.040	0.294	0.453	2.205
Junior high school	−0.28 (−0.850, 0.271)	−0.045	0.311	0.337	2.967
Polytechnic or senior high school	−0.55 (−1.241, 0.124)	−0.067	0.109	0.381	2.622
College and above	−0.49 (−1.392, 0.410)	−0.039	0.285	0.503	1.988
Occupation					
Ordinary staff (reference)					
Professionals	−0.21 (−0.888, 0.458)	−0.029	0.531	0.317	3.159
Manager	−0.15 (−1.408, 1.100)	−0.007	0.810	0.749	1.335
Service industry employee	0.09 (−0.715, 0.913)	0.009	0.812	0.445	2.248
Production staff	−0.09 (−0.757, 0.564)	−0.016	0.774	0.205	4.872
Others	−0.49 (−1.359, 0.368)	−0.043	0.260	0.454	2.200
Financial strain					
Sufficient (reference)					
Approximately	0.21 (−0.152, 0.578)	0.036	0.252	0.679	1.473
Difficult	0.98 (0.438, 1.533)	0.126	0.000	0.514	1.947
Harmonious family					
Disharmonious (reference)					
Harmonious	−1.52 (−2.383, −0.657)	−0.107	0.001	0.678	1.476
Filial piety	−0.36 (−0.583, −0.146)	−0.100	0.001	0.707	1.415
Self-rated health					
Bad (reference)					
Fair	−0.691 (−1.202, −0.181)	−0.111	0.008	0.375	2.666
Good	−1.306 (−1.826, −0.786)	−0.221	0.000	0.324	3.083
Suffering from chronic diseases					
No (reference)					
Yes	0.378 (−0.064, 0.821)	0.047	0.094	0.835	1.197
Functional impairment	0.070 (0.005, 0.136)	0.060	0.036	0.800	1.250
Life satisfaction					
Dissatisfaction (reference)					
Fair	−2.651 (−3.397, −1.904)	−0.280	0.000	0.406	2.462
Satisfaction	−4.259 (−4.921, −3.597)	−0.565	0.000	0.327	3.056
Constant	11.923 (9.596, 14.251)	-	0.000	-	-
Adjusted R Square	0.482				
*F*	33.050				
*p*	0.000				

## Data Availability

The data presented in this study are available upon request from the corresponding author. The data are not publicly available due to privacy restrictions.
